# Case of Suspension of the Mental Faculties

**Published:** 1846-02

**Authors:** 


*Case of suspension of the mental faculties, of the powers of speech, and special senses,* with the exception of sight and touch, occurring in a young woman, and continuing for many months, in consequence of her having accidentally fallen into a river, and being nearly drowned. By ROBERT DUNN, Esq., Surgeon.—Many and curious are the instances which have been recorded of the arrest, suspension, and, at the time, apparent obliteration of the most ordinary functions of the nervous system, in consequence of a sudden shock, agitation or fright.

Such cases are not more startling to the minds of common observers, than they are interesting to the physiologist and medical inquirer. The following plain narrative of an interesting case, which has come under my observation, may not be unworthy of the notice of my professional brethren. The subject of the narrative is a young woman, now in the nineteenth year of her age, of a mixed temperament, the *nervous* with the *lymphatic*, and, as the medical attendant of the family, I have had frequent opportunities of seeing her since she was two years old. She was the first child of her parents, and came prematurely into the world, about six weeks before the usual term of gestation. No reasonable hope was at first entertained that she could live. She had not strength to suck, but the milk, of which, fortunately, there was an ample supply, was drawn from the breast, and she was fed with it by a spoon, and from a nursing bottle. At the end of six weeks, the completion of the reputed time of gestation, she was in convulsions for nearly an entire day, and all hope of her living was extinguished; but, nevertheless, she did survive, and from this time she began to thrive, to the great astonishment of every one who had seen her. She improved rapidly, and when, at four years old, she was inoculated for the small-pox, she was a fine, strong, and healthy child, stouter and larger than most children at her age. A custom prevailed in the village to which she was removed, and where, indeed, she had been born, that whenever the *small-pox broke out naturally*, as it was termed, among the inhabitants, the other children of the village should be *inoculated* if their parents or friends thought proper to have it done. At other times, inoculation was not permitted. She was, accordingly, inoculated, and had the small-pox so severely that it nearly cost her her life. She passed favorably through the other ordinary diseases of childhood, with the exceptiou of scarlatina, which she had in a severe form when she was ten years old. After the attack of scarlatina, she enjoyed uniformly good health, up to the date of the present narrative. and grew up a strong, robust, and hearty young woman. Her perceptive powers were naturally quick, and, being largely endowed with the imitative faculty, she was put to the business of a milliner and dress-maker. Two years ago, her health beginning to suffer from long and close application to business, she went, on my recommendation, to her grandfather's, in the county of Kent, and, while there, the following accident occurred:

On the 14th of July, 1843, as she was walking with her aunt, by the side of the river, which runs through the park of Sir Percival Dyke, of Lullington, she accidentally let a parcel which she was carrying slip from her hands, and in the effort to save it fell into the water. She laid hold of the grass upon the side of the bank, but this giving way, she again fell backwards into the water. Her aunt immediately ran for assistance, but on getting back nothing was to be seen, but, about twenty yards below the place where the accident had happened, a part of the girl's shawl was observed to be floating upon the surface of the water; and from this place she was eventually dragged, in about a quarter of an hour from the time of her falling in, in a state of suspended animation. She was carried to the house of Sir Percival Dyke, where she received every attention. A medical man was sent for, and after the lapse of about six hours, she became sensible, and so far recovered as to be well enough to be removed, on the afternoon of the following day, to her grandfather's house at Shoreham. Though she was now sufficiently recovered to give some account of the accident, and of the state of her feelings after falling in, on her first attempt to get out, she was far from well, complaining of great uneasiness about the bowels, and of pain in the head. The bowels were obstinately constipated; there was no evacuation, from the day of the accident until the 20th of the month, a period of seven days; and when the constipation gave way, the alvine dejections proved that both mud aud gravel had passed into the stomach. On the 24th, she was seized with a fit (up to this time she had been quite sensible,) and is described as lying in the fit in a state of complete stupor for nearly four hours, when she opened her eyes, but was deprived of the powers of speech and hearing, and of the senses of taste and smell; her mental faculties quite benumbed or paralyzed, giving no indication that she recognized any of her friends about her. The head was shaved, and covered with ice; a blister applied to the nape of the neck, and other remedial measures adopted. I regret that I cannot furnish more precise information as to the nature of the fit, and of the treatment pursued. I have written twice to Mr. Richards, on the subject, but have not been favored with any reply to my notes.

After the lapse, however, of about three weeks, she was so far recovered as to be able to be removed to London; and I saw her, for the first time after the accident, at her own home, on the 8th of August. She did not, or rather could not, recognize me, for her physical faculties were quite suspended; indeed, her only medium of communication, at this time, with the external world was through the senses of *sight* and *touch*, for she could neither *hear nor speak, smell nor taste.* Her vision, at short distances, was quick, and so great was the state of exaltation of the general sensibility upon the surface of the body, that the slightest touch would startle her; still, unless she was touched, or an object and person was so placed that she could not avoid seeing the one or the other, she appeared to be quite lost to everything that was passing around her. She had no notion that she was at home, nor the least knowledge of anything about her; she did not even know her own mother, who attended upon her with the most unwearied assiduity and kindness. Her memory, and the power of associating ideas, were quite gone. Wherever she was placed, there she remained throughout the day. She was very weak, but her bodily health was not much deranged; the tongue was clean; the skin moist; and the pulse quiet and regular; but the bowels sluggish. At the time of the last menstrual period there had been considerable general irritability, attended with some febrile disturbance, and with an increase of heat about the head, and these symptoms I noticed on subsequent occasions. The catamenia had followed immediately upon the accident; it re-appeared at the proper time, and continued regular and copious throughout her illness. Her appetite was good, but having neither taste or smell, she ate alike indifferently whatever she was fed with, and took nauseous medicines as readily as delicious viands. She required to be fed; when I first saw her, she had no notion of taking food that was placed before her, but, a few days afterwards, if a spoon was put into her hands, and filled by her mother, and conveyed for a few times to her mouth, she would afterwards go on by herself until the whole was eaten.

Her wants were sedulously attended to, but she manifested no uneasiness as to food, however long she might be kept without it. The same thing was observed in reference to drink. The calls of nature were alike unheeded by her; the urine and fæces were voided *unconsciously*, but with the striking peculiarity, that, during the expulsion of fæces, such was the *reflex action* induced that the extremities became spasmodically convulsed and rigid; the head was thrown backwards; the muscles of the neck were stiff; and the eyelids closed; so that her mother considered that her bowels never acted without her having what she called "a convulsion fit;" the same thing occasionally happened when the bladder expelled its contents, and what is still more remarkable, the same tonic rigidity of the muscles invariably took place whenever she went to sleep. Every night when she was placed upon the bed, she remained for some time in a semi-recumbent posture, after which the eyelids closed and the head fell backwards upon the pillow; the hands were clenched; the muscles of the neck stiff; the arms and legs in a state of tonic rigidity, the latter always crossed the one upon the other; after a time, the muscles became relaxed; she turned upon her side, and slept soundly until the morning.

Such was the state in which I found the young woman on her arrival in London, nearly one month after the occurrence of the accident, and I may here anticipate the narrative by stating that from the time her mental faculties became suspended, in the fit on the 24th of July, 1843, until the July of the following year, when they were again restored—her life, to herself, is *one continued blank*. She has not the slightest knowledge or remembrance of anything which took place during the interval. I put her at once upon a course of tonic medicine, giving her of the compound mixture of iron ℥iss.

three times a day, and a frequent aperient of the aloes and myrrh-pill, adding, occasionally, a few grains of blue pill.

One of her first acts on recovering from the fit had been to busy herself in picking the bed-clothes, and, as soon as she was able to sit up and to be dressed, she continued the habit by incessantly picking some portion of her dress; she seemed to want an occupation for her fingers, and, accordingly, part of an old straw bonnet was given to her, which she pulled to pieces of great minuteness; she was afterwards bountifully supplied with roses; she picked off the leaves, and then tore them into the smallest particles imaginable. A few days subsequent she began forming upon the table, out of these minute particles, rude figures of roses and other common garden flowers; she had never received any instruction in drawing.

Roses not being so plentiful in London, waste paper and a pair of scissors were put into her hands, and for some days she found an occupation in cutting the paper into shreds; after a time these cuttings assumed rude figures and shapes, and more particularly the shapes made use of in patch-work. At length she was supplied with the proper materials for patch-work, and, after some initiatory instruction, she took to her needle, and in good earnest to this employment. She now labored incessantly at patch-work from morning till night, and on Sundays and week-days, for she knew no difference of days nor could she be made to comprehend the difference. She had no remembrance from day to day of what she had been doing on the previous day, and so every morning commenced *de novo*. Whatever she began, that she continued to work at while daylight lasted, manifesting no uneasiness for anything to eat or to drink, taking not the slightest heed of anything which was going on around her, but intent only on her patch-work. Occasionally, indeed, and not unfrequently two or three times in the course of the day, she would have what her mother called her "fits." Whilst intent upon her work, and without any external exciting cause, her head would fall backwards, her eyelids close, her arms and legs become rigid, and her hands clenched. After a short time, varying in extent from a few minutes to half an hour or more, the muscles would become relaxed, the eyes open, and she would resume her work, apparently unconscious that anything had happened. About this time she began to show indications of feeling interested in the figures of the flowers and buds, &c., upon the silk and other materials which are made use of in patch-work. The perception of colors, and the exercise of the imitative faculty, were the first evidences she exhibited of physical advancement in her present state. Although she had received a good plain education, and had been very fond of books, now she could neither read nor write, nor even be made to comprehend the letters of the alphabet. All her former knowledge and past experience appeared to be obliterated, or at least, for the time, to be buried in oblivion, with one exception—a feeling of dread or fright in connection with water; and she now began, *de novo*, like a child, to acquire ideas and to register experience. Admitting that the senses are the only inlets of all the materials of knowledge, and that—"Nihil est in intellectu quod non prius fuerit in sensu," it was not to be expected when in this abnormal condition, with only the senses of sight and touch in communion with the external world, that her progress could be otherwise than slow in the extreme. However, she evinced an interest in looking at pictures and prints—more especially of flowers, trees, and animals—but when shown a landscape in which there was a river, or the view of a troubled sea, she became instantly excited and violently agitated, and one of her fits of spasmodic rigidity and insensibility immediately followed. If the picture were removed before the paroxysm had subsided, she manifested no recollection of what had taken place, but so great was the feeling of dread or of fright associated with water that the sight of it in motion, its mere running from one vessel to another, made her shudder and tremble, and in the act of washing her hands they were merely placed in the water. After she had been at home a fortnight, I had the benefit of frequent consultations with my friend, Dr. Todd, of King's College, on her case She had partially recovered her taste, and refused any longer to take nauseous medicines. Dr. Todd expressed his conviction that eventually she would perfectly recover, however gradual and protracted the recovery might be—an opinion which has been fully verified. The accident happened in 1843, and it is only within the last few weeks that she has completely regained her hearing, and the full exercise and enjoyment of her mental and bodily powers. We agreed that the tonic course of medicines should be continued, combining quinine with the iron. We further advised the frequent sponging of the head and spine with cold water, and the use of the shower-bath, as soon as it could be borne. The first attempt, in accordance with onr wishes, of running water through a common cullender upon her head, induced such alarming excitement and fright, and was followed by a fit of insensibility of such long continuance, that the experiment was not repeated.—*London Lancet*,

